# Supplementary figures and images for: High-resolution transcriptional impact of AIRE: effects of pathogenic variants p.Arg257Ter, p.Cys311Tyr, and polygenic risk variant p.Arg471Cys
Source: Front Immunol. 2025 Apr 22;16:1572789. doi: 10.3389/fimmu.2025.1572789 (PMC12053179; doi:10.3389/fimmu.2025.1572789)

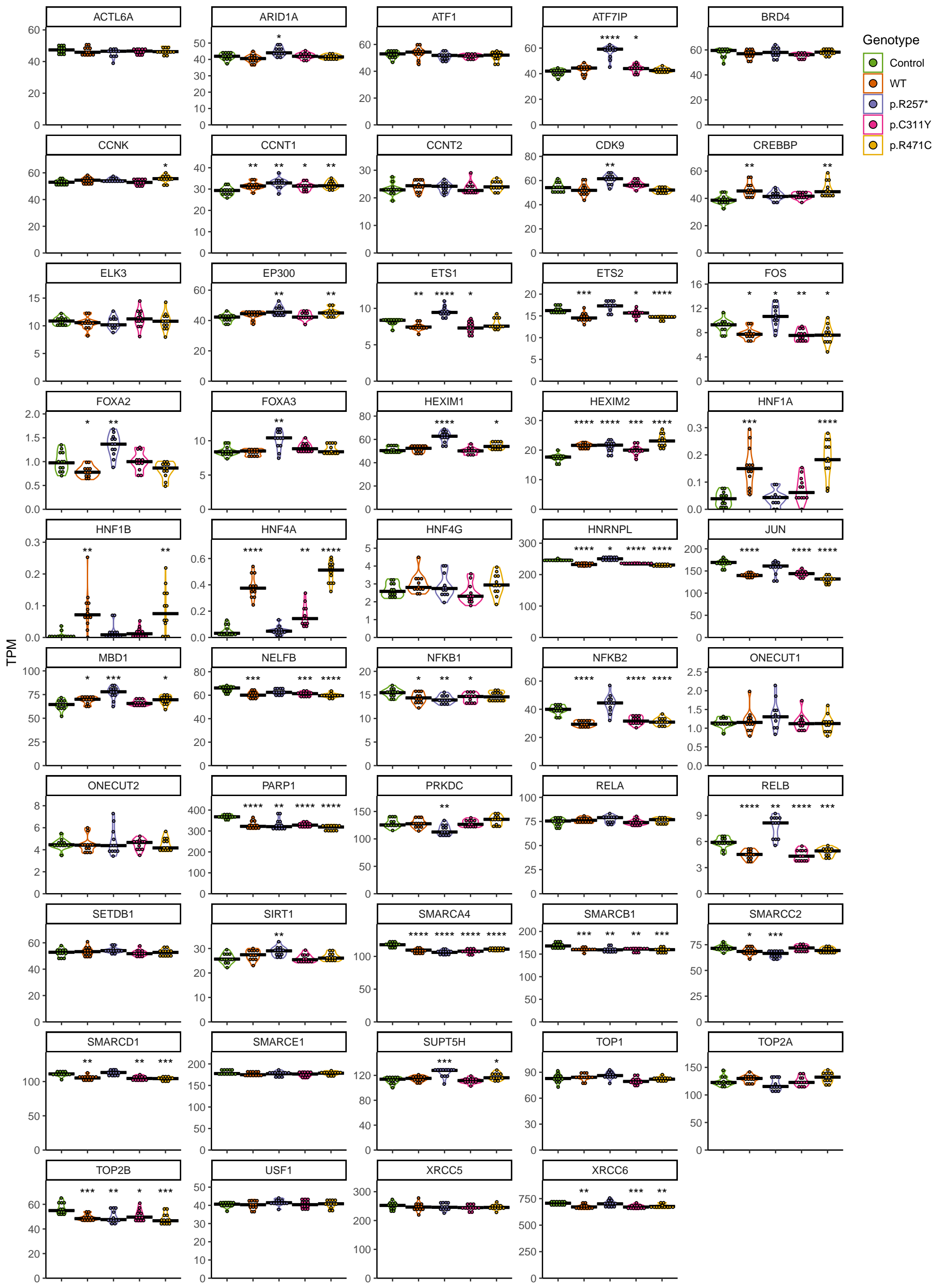

Supplement: Supplementary file 1 [file DataSheet1.pdf]
